# In Their Own Words: How Opioids Have Impacted the Lives of “Everyday” People Living in Appalachia

**DOI:** 10.13023/jah.0204.05

**Published:** 2020-09-01

**Authors:** Patricia N. E. Roberson, Gina Cortez, Laura Hunt Trull, Kathrine Lenger

**Affiliations:** University of Tennessee, Knoxville; University of California Davis; James Madison University; University of Tennessee, Knoxville

**Keywords:** Appalachia, opioids, substance abuse disorder, Remote Area Medical Clinics, culture, prevention, rural health, drug use

## Abstract

**Introduction:**

The opioid epidemic is ravaging people, families, and communities in Appalachia. However, limited research has examined how “everyday” people (e.g., not chronic pain patients, not medical professionals) living in these communities how opioids have impacted their lives.

**Objective:**

Identify the perception of the opioid epidemic on individuals, families, and communities from people living in region most impacted regions.

**Methods:**

Patients were recruited at Remote Area Medical clinics throughout Central and Southern Appalachia to complete interviews online (N = 169) or over the phone (N = 26), including one open-ended question about how opioids have impacted their lives.

**Results:**

Using the qualitative method content analysis, several themes were identified, including both the positive and negative impact of opioids from the online interviews. Additionally, resiliency was found to be a common theme and a theme not often emphasized by scholars and the media. These themes also highlight the importance of social support in these communities. Further, in the phone interviews, we were able to replicate the themes, and an additional theme was identified: Systemic Cause of Opioids.

**Conclusion:**

Opioid intervention must be comprehensive and include the cultural context that recognizes community ties, family and kinship support, resilience, and systemic barriers to addressing the opioid epidemic. Future interventions must harness the existing resiliency and social support in these communities to effectively combat the opioid crisis in Appalachia. Otherwise, opioids will remain the insider and further insulate Appalachian communities from systemic recovery.

## INTRODUCTION

People who are underserved by health care tend to be lower-income or living in rural areas. These underserved people are affected disproportionately by the contextual factors that affect health, such as the prevalence of food deserts, shortage of healthcare professionals, economic depression, and often high rates of opioid misuse.^1^ These contextual factors have been especially true in Appalachia, the area of the country known for deep cultural ties and poverty.^2^

Appalachia consists of counties in Alabama, Georgia, Kentucky, Maryland, Mississippi, New York, North Carolina, Ohio, Pennsylvania, South Carolina, Tennessee, Virginia, and West Virginia. Seventy percent of Appalachian counties are health professional shortage areas,^3^ and half of the states have not expanded Medicaid to include lower-income adults.^4^ In Appalachia, opioid misuse is associated with higher poverty, 37% higher drug overdose deaths; the region has been identified as a high-intensity drug trafficking area.^5^ The combination of high healthcare needs and inadequate service provision has been overwhelming for Appalachia. Further, changes in the energy economy around coal and increases in opioid misuse have increased healthcare needs, yet service provision has not responded in part.^6^

Despite the devastating impacts of opioid misuse and economic downturns, Appalachian people have a strong and proud identification with rural culture. Rural culture is multifaceted and includes variations in demographic, economic, or social factors.^7^ People living in rural communities tend to have a more traditional culture,^8^ have a keen sense of shared culture and social cohesion,^7^ and are more religious.^9^ These individuals also have a high regard for independence and self-reliance^7^ while simultaneously relying on family and social networks for healthcare advice and recommendations.^10^ There is evidence that rurality is related to health outcomes, both positively and negatively.^11^ For example, rural patients tend to respond well to health treatments, especially with more proactive individual characteristics such as health self-efficacy.^11^,^12^ Still, they have higher rates of comorbidities and mental health concerns.^11^ The majority of our sample is from these rural communities in Appalachia.

Most of the research on the opioid crisis in Appalachia focuses on chronic pain patients or opioid users.^13^–^18^ Despite the breadth of issues covered by these patient-focused studies, they cover only individuals who actively use opioids (either as prescribed or for recreation) or individuals in recovery. This body of research does not focus on “everyday” individuals who may have been affected by the opioid crisis in their community.

### Theoretical Framework

High poverty, high drug use, high overdose rates, low education levels, and economic struggles are well-documented circumstances that plague Appalachia.^5^,^18^ Yet, the Appalachian people press on and possess strengths, pride, and kindness unseen in other parts of the nation.^19^ This study is grounded in Community Resilience Theory, which focuses on the community’s ability to adapt to a stressor; in this case, opioid misuse that has led to the current opioid crisis. The community responds to the crisis by resisting and adapting, demonstrating community resilience, and moving toward positive outcomes.^20^ This theory informed the wording of questions and methods of analysis as the research focuses on amplifying the strengths by which Appalachian people continue to persist and have hope for their communities.

In this study, we sought to understand the impact of the opioid crisis on underserved individuals, families, and communities from the perspective of “everyday people” living in the Appalachian rural, lower-income regions, which have been affected most by the opioid crisis.

## METHODS

### Procedures

Participants were recruited from Remote Area Medical (RAM) clinics across seven clinics in Central and Southern Appalachia. Participants completed surveys over the telephone or online. The online and telephone samples were asked the same stem question: *We’ve asked you a lot of specific questions. But we want to know, in your own words, how have opioids affected you, your family, and your community? There is no wrong answer, we just want to know your experience*. A detailed description of the procedures is provided in the Additional Files.

### Analytic Plan

#### Content Analysis

Qualitative survey responses were analyzed using content analysis methods. Two of the authors served as multiple analysts to develop themes; initial coding was done on paper using consensus coding. Content analysis was conducted by pen and paper as the authors were able to spend time together with the data for analysis purposes and combine the online and phone surveys into a paper document. Credibility was established with the triangulation of more than one interview (initial and follow-up) as well as multiple analysts.^21^ The third author strengthened the methods and analysis by serving as a peer to debrief and to validate the concepts assessed.^22^ An audit trail was maintained throughout in a Word document to detail how the information was collected, and thematic connections were drawn from participant narrative.^23^

## RESULTS

### Participants

Participants were mostly from rural regions, were mostly white, and about half reported having a high school education or less. A more detailed description of the telephone and online samples are provided in the Additional Files.

### Content Analysis Results

From the online and telephone data, several themes emerged across varying levels of society: (1) individual impacts of opioids, (2) family impacts of opioids, and (3) community impacts of opioids. Table 1 provides an overview of themes and subthemes that are discussed more thoroughly in the Additional Files.

### Study 1: Online interview

Using the content analysis method, three overarching themes were identified in the interview responses: (1) benefits of opioid use, (2) negative impact of opioids, and (3) resiliency. Highlighted within each theme are several subthemes, how themes often span multiple levels of society (i.e., individual, family, community), and the similarities in the participant’s responses. It is important to note that, at times, responses fell into more than one category.

### Study 2: Telephone Interview

Also using the content analysis, Study 2 identified similar overarching themes with the addition of a fourth theme (Systemic Restrictions and Frustrations). Despite these similarities in theme categories, the telephone interview provided more nuanced detail about the participants’ perspective. This is likely because it is often easier to talk then type responses to open-ended questions. These four themes from the telephone interviews were: (1) benefits of opioid use, (2) negative impacts of opioid use, (3) resiliency, and (4) systemic restrictions and frustration.

## DISCUSSION

Through content analysis, several categories emerged across four themes and several subthemes. Rural and underserved participants in this study have a clear awareness of the opioid epidemic in rural areas and the positive and negative impacts at the individual, family, and community levels. They also exhibit several qualities of resilience at the individual and family level with keen observations about the systemic implications in their communities and the nation. Generally, these themes were partially reflected in the previous literature but from a different and important perspective of “everyday” people living in Appalachia rather than from the perspective of chronic pain patients or physicians. One newer theme within the sample was resiliency, which was not frequently highlighted in the previous qualitative literature focusing on medical professionals, individuals using opioids as prescribed, or using it recreationally.

The first theme was about the perceived benefits of opioid use. Some participants stated that prescription opioids were beneficial to them and loved ones. Appropriate use of opioids improved participants’ quality of life for those with chronic pain. Within this theme were underlying frustrations with programs and policies that limited their access to opioid prescriptions among the online sample. This underlying frustration has indeed been observed in the literature previously.^24^ However, what was not mentioned among our participants is that this frustration toward limited access to opioid prescriptions also strains the patient–physician relationship reducing trust from medical professionals^18^,^24^ and reducing physicians’ trust in their capacity to monitor patients’ opioid use.^25^,^26^ This potentially creates a negative feedback loop of patient–physician interaction reducing access to opioids for patients who need them for their quality of life. However, negative attitudes physicians may have toward people who have on opioid disorder can shift with education.^27^ Perhaps increased physician education around identifying opioid misuse can improve long-term patient–physician relationships and improve physician trust in patient usage in order to ultimately improve access for those patients who need opioids for functioning and quality of life.

What has less frequently been discussed in the literature on the opioid epidemic for rural and underserved communities, specifically Appalachia, is resiliency. This concept was present throughout the data and emerged as an important category that informed the theme of resiliency. The qualitative data collection and analysis were essential to building a narrative around the recovery from the opioid epidemic *as a community* and *as a culture* for Appalachia. Previous research has told the contrary story that rurality is more often espoused as a risk factor for opioid misuse and abuse.^28^ While Thomas and colleagues^29^ allude to the social networks being a potential protective factor, they more often emphasize the risk associated with social networks, including lack of knowledge about treatment and risk behaviors. Additionally, Yedinal and colleagues^30^ negatively identified social networks for young people as the primary way through which initial opioid misuse began, social gatherings as places where drug mixing and incidents of unintentional overdose were common. However, based on the current findings, individuals’ resiliency to opioids is through the support of their social network—parents moving their families, relatives caring for users’ children. While social networks, no doubt, can increase the risk of opioid misuse through access and encouraging increased usage, the previous literature does not highlight the critical role of a social network in improving resiliency. Future intervention to reduce the risk of opioid misuse should focus on strengthening social networks, shifting misinformation circulating in social networks about treatment options, and harnessing the power of social networks to reduce stigma around opioid misuse to build on the resiliency already present in Appalachian communities.

### Limitations

This study is not without limitations. First, the participants in this study were not randomly selected; it was a convenience sample from patients who attended a safety-net clinic. Therefore, the themes identified may be biased by the participants who volunteered for this study. While Remote Area Medical serves the most underserved individuals in the communities it reaches, permission was not given to survey participants on site. Meaning that those who participated had to have access to a telephone to complete this study. This likely means that there was no access to the opinions of the most underserved Remote Area Medical participants, including homeless, individuals without consistent access to telephones, or individuals in the most rural communities without telephone access. However, these findings are a first step in understanding how “everyday” people in Appalachia perceive the impact of the opioid clinic on themselves, their families, and their communities.

## CONCLUSION

This research stands to inform future practices for community-level interventions in the opioid epidemic in Appalachia and other rural areas. The media continually emphasizes the statistics of rural poverty, blight, and poor health outcomes.^31^ Based on the results of this study focusing on “everyday” individuals living in these communities, we have captured an important component that can be utilized when intervening on the opioid crisis—resiliency and social support. Evidence-based practices are only recently beginning to be tested in rural areas or with rural people who exhibit distinctly different cultural contexts and social conditions as compared to their rural counterparts. Interventions have targeted *individual* treatment and/or avoidance strategies such as removing children from homes where a parent is abusing opioids. While these strategies are important and necessary, they lack the comprehensive cultural context that recognizes community ties, family and kinship support, resilience, and systemic barriers to addressing the opioid epidemic. Future interventions must take the existing resiliency and social support into account to be effective in combatting the opioid crisis in Appalachia. Otherwise, the opioids will remain the insider and further insulate Appalachian communities from systemic recovery.

**SUMMARY BOX**

**What is already known about this topic?** The opioid epidemic is ravaging people, families, and communities in Appalachia. Medical professionals, chronic pain patients, and recovering drug users have been frequently studied in qualitative studies about the impact of the opioid epidemic.

**What is added by this report?** We target “everyday” people to understand their perspective about the impact of the opioid epidemic on their lives, their families’ lives, and broadly the impact on their community. We find that participants see both positive and negative impacts of opioid use and several participants identify resilience.

**What are the implications for future research?** Many opioid interventions lack a comprehensive cultural context that recognizes community ties, family and kinship support, resilience, and systemic barriers to addressing the opioid epidemic. Future interventions must harness the existing resiliency and social support in these communities to effectively combat the opioid crisis in Appalachia. Otherwise, opioids will remain the insider and further insulate Appalachian communities from systemic recovery.

## LITERATURE REVIEW SUPPLEMENT

There is a substantial body of qualitative research that explores health providers’ perspectives on the impact of the opioid crisis. Many of these studies focus on how the opioid crisis shapes professionals’ prescribing patterns.^1^,^2^ However, when physicians begin to stop prescribing, potentially because they suspect addiction, opioid users have a higher tendency to visit emergency departments, inpatient hospitals, and outpatient medical clinics (e.g., safety-net clinics like Remote Area Medical [RAM]), rather than revisiting their primary care physician.^3^ Studies on physicians that volunteer at safety-net clinics have shared similar sentiments when prescribing opioids as the previously noted studies that there is a fear of harming patients, the community, and opioid addiction and overdose.^4^ Patients in this study also noted increased difficulty in getting opioid prescriptions due to policies aimed at mitigating opioid misuse.^4^ While this research is essential to understanding how healthcare systems may be linked to opioid misuse, it does not target the lived experiences of people living in Appalachian communities where opioid misuse is pervasive.

Most of the research on the opioid crisis in Appalachia focuses chronic pain patients or opioid users. For example, one study, focusing on people who use opioids recreationally, found fears of stigma and law enforcement lead users to change their usage setting to one that increased the risk of overdose.^5^ Antoniou and colleagues^6^ found that, from the perspective of people who misuse opioids, harm reduction policies could be problematic because they propagated the stigma of opioid use, made the individuals feel a loss of autonomy, and exacerbates the existing vulnerabilities of poverty and drug criminalization. Similarly, Allen and colleagues found that patients believed that suspending syringe service programs increased the perception of stigmatization and decreased access to naloxone and routine HIV testing.^7^ Another study focused on individuals in recovery, trying to reenter the workforce.^8^ These individuals emphasized how finding a new identity, lifestyle, and purpose is essential with their recovery, and that work can be an important part of that rediscovery.^8^ Furthermore, in a study partially targeting addiction clinic patients’ perceptions on the opioid crisis found several themes including: prescription drug abuse access being connected with legitimate and illegal routes, both being quickly accessible, and that the underlying rationale for acquiring and illegally distributing prescription drugs for abuse was both increased tolerance or addiction to the pain medication and that the distribution of these prescription drugs was a source of revenue.^9^ These themes provide important information about individuals’ perceptions about why and how opioid abuse occurs in their community. For chronic pain patients, there appear to be several ongoing struggles in maintaining pain medication use, but they had to “just keep plugging.” This suggests a fortitude to manage the chronic pain they have despite their frustrations with logistic barriers at treatment facilities.^10^

## METHODS SUPPLEMENT

Quantitative studies of opioid use dominate the research on the impacts of the opioid epidemic. However, qualitative studies are well suited for revealing how circumstances play out in a particular context. In this case, underserved Appalachian culture (i.e., rural communities or people living with a lower income) is the context for the study, and investigating the phenomenon in this way will provide more depth of understanding.

### Procedures

Participants were recruited from RAM clinics across seven clinics in Central and Southern Appalachia. RAM is an organization that has been providing medical, dental, and vision clinics in rural, underserved, and remote areas since 1985. They are headquartered in Appalachia and provide approximately half of their clinics in the Appalachian region. RAM provides a highly valued service – people who would not ordinarily be able to have their teeth repaired or extracted, who may not be able to purchase glasses or who lack access to any preventative care such as mammograms or vaccinations, can get these services with no questions asked. The very poor and uninsured, often immigrants and homeless, wait in line for days to receive these healthcare services. In areas that have been so heavily struck by the opioid epidemic, there are a substantial impact among these, particularly vulnerable patients.

We used a convenience sample of RAM clinic participants. During the clinics, as many patients as possible were given postcards with the advertising of the volunteer opportunity to participate in research to better understand the patients who attend RAM clinics. A researcher was stationed in the clinic traffic flow strategically to catch as many patients as possible, but an unknown number of patients may not have been recruited. Patient recruitment was completed at seven clinics across Appalachia between October 2018 and June 2019. Participants could participate in the study if they attended the clinic and were 18 years or older. Upon recruitment, participants were given informed consent. Participants were mailed a $10 gift card after completing the survey. We estimate that approximately 5% of individuals who were given a postcard were recruited into the study and completed the survey; we derived this number by the total number of individuals who completed the survey divided by the total number of postcards given out across the clinics. The postcard asked interested individuals to text or call the research specific phone number within one week of the clinic. Participants who texted the phone number completed the survey and relevant qualitative questions in an online written survey. Participants who called the phone number completed the survey and relevant qualitative questions in an oral telephone interview that was recorded. In total, 189 participants consented and completed all parts of the survey included in this study. Because of the different methods for completing the survey, the set of data will be addressed separately in sequential studies: Study 1 (online interview sample) and study 2 (telephone interview sample). This study was approved by the University of California, Davis Institutional Review Board (IRB).

The online and telephone samples were asked the same stem question: *We’ve asked you a lot of specific questions. But we want to know, in your own words, how have opioids affected you, your family, and your community? There is no wrong answer, we just want to know your experience*. For the online interviews, there were no follow-up questions due to the nature of the data collection. For the telephone surveys, interviewers were trained to ask follow-up questions to get the participants’ opinions on how opioids had affected all three aspects: themselves, their family, and the community. In addition to demographic questions, participants were asked whether they identified as urban, suburban, or rural. Rurality is defined in the literature in a variety of ways, often based on the zip code where the participant resides. In this case, it is more effective to allow participants to self-identify as rural for several reasons. First, participants in our sample were often transient and may not have a residence or identify with a particular ZIP code. Secondly, while they may have a ZIP code where they currently reside, that residence could be a shelter or staying with a friend in a more urban area that is not consistent with their cultural identity of rural. Rural culture is an important and distinct component of identity, and self-report is the most accurate way of determining rurality in this qualitative study in addition to being an emerging strategy for determining rurality in the literature.^11^

### Analytic Plan

#### Content Analysis

Qualitative survey responses were analyzed using content analysis methods. Two of the authors served as multiple analysts to develop themes. Initial coding was done on paper using consensus coding between the authors. Content analysis was conducted by pen and paper as the authors were able to spend time together with the data for analysis purposes and combine the online and phone surveys into a paper document. We established credibility with the triangulation of more than one interview (initial and follow-up) as well as multiple analysts.^12^ The third author strengthened the methods and analysis by serving as a peer to debrief and to validate the concepts assessed.^13^ An audit trail was maintained throughout in a Word document to detail how the information was collected, and thematic connections were drawn from participant narrative.^14^

#### Researchers

The researcher’s personal goals, previous knowledge, and assumptions can affect the development of the study and the analysis. The first author has a doctoral degree in Child and Family Studies and is currently faculty in a College of Nursing. Family studies researchers typically operate from a systems theory perspective whereby the underlying assumption is that all systems within a community and all individuals within a family are interconnected. This author has volunteered at over 20 Remote Area Medical clinics and is experienced working directly with the population sampled in this study. Further, the majority of this author’s research focuses on underserved communities including low-income and rural families in the Appalachian region. The second author is a master’s level social worker with a doctoral degree in nonprofit and community leadership. Social workers operate from a strengths-based perspective rooted in systems theory. These authors’ areas of interest are in rural health disparities, health and social policy, and community engagement. This author has volunteered at over 15 Remote Area Medical clinics, is very experienced working with the population, and is interested in the topic as a resident of a county bordering Appalachia. The final two authors are trainees of the first author.

#### Data

Undergraduate research assistants transcribed telephone interviews. The online survey responses and telephone transcriptions were put into separate excel databases for coding. Responses were read through in their entirety before coding began.^15^ The authors completed the initial coding cycle independently and identified themes of each participant’s response. Next, the authors met as a group four times to refine our categories and arrive at consensual themes. The refining of themes was done to select the most salient overall categories representing the participants’ experiences of how the opioid crisis has impacted individuals, families, and communities.

## RESULTS SUPPLEMENT

### Participants

The online interview sample had 169 participants. Of this sample, 60% of participants reported living in a rural area, 22% in a suburban area, and 17% live in an urban area. The majority reported being a woman (66%) and having children (62%). In terms of race/ethnicity, the vast majority identified as White (89%), followed by 5% who identified as Black, and the remaining 6% identified as another race/ethnicity. In terms of education, 51% reported having a high school education or less, 38% reported having some college, and 11% reported having a college education or a graduate degree. On average, participants were 36.54 years old (*SD* = 11.81. range = 18–73). Fifty-nine percent report having some type of health insurance.

For the telephone interviews, 26 individuals participated. The majority of this sample reported having some type of health insurance (72%). The majority of this sample also reported being a woman (73%) ad having children (68%). In terms of race/ethnicity, 89% identified as White, 6% identified as Black, and the remaining 5% identified as another race/ethnicity. In terms of education, 57% reported having a high school education or less, 32% reported having some college, and 11% reported having a college education or a graduate degree. On average participants were 47.17 years old (*SD* = 16.64. range = 18–88). Fifty-six percent of these participants reported living in a rural area, 14% in a suburban area, and 31% live in an urban area.

### Content Analysis Results

From the online and telephone data, several themes emerged across varying levels of society: (1) individual impacts of opioids, (2) family impacts of opioids, and (3) community impacts of opioids. See figure 1 for an overview of themes and subthemes that we discuss more thoroughly in the supplemental file online.

### Study 1: Online interview

Using the content analysis method, we identified three overarching themes in the interview responses. These themes were: (1) Benefits of Opioid Use, (2) Negative Impact of opioids, and (3) Resiliency. Within each theme, we highlighted several subthemes, how themes often span multiple levels of society (i.e., individual, family, community), and we took note of similarities in the participant’s responses. It is important to note that, at times, responses fell into more than one category.

### Benefits of Opioid Use

In our first theme, several participants expressed the perception of the impact of opioids on themselves, their family, and their community as positive, despite the current opioid epidemic. Of the 169 participants, 36 participants’ responses fell into this first theme. In the responses, the data showed that some participants believe that, when not abused, opioids can be beneficial to those in severe pain and need. For example, one participant explained how opioids positively impact them and their family:

*They absolutely helped with headaches. 100% they do. They also cause stomach problems and weight gain…for family, they don’t abuse it, and it really helps them. They don’t have any problems*.

This participant reported that opioids are not a problem for themself or their family. While they acknowledge adverse side effects that came with using opioids, because they were using responsibly and they were benefiting (i.e., no more headaches), they did not see an issue with opioids for them self and their family.

### Negative Impact of Opioids

The second overarching theme summarized many of the participants’ frustration with the negative impact of opioids in their community, family, and at times themself. Of the 169 participants, 85 participants expressed one subtheme of this theme.

#### Negative Impact of Opioids: The Limitation of Access to Opioids

One subtheme was *the limitation of access to opioids*. Some participants explained how systemic changes caused by the opioid epidemic impacts their access to legally prescribed opioids, affecting the quality of life for their loved ones or themself:

*See, my husband has pain levels of a woman having child-birth 24 hours a day. He was on opium (sic), and they took him off it because of the opioid problem, and they won’t give [him] anything. I think this is a stupid thing. I don’t agree with the opioid problem*.

This participant’s husband was in an accident in the past and uses a wheelchair and suffers from severe back pain. This participant reported that they were negatively impacted by the opioid epidemic in that they no longer had access to their prescribed opioids. In a similar sentiment, another participant expressed frustration about no longer having access to opioids:

*Without opioids, I wouldn’t be able to do things for my family. They work. However, I live in an area where they are abused. Because of this, I can’t get them anymore. I have severe sciatic nerve pain. It’s the only thing that had worked for me*.

This participant evidenced how particular areas are becoming stricter on opioid prescriptions, despite people who were currently using opioids reporting responsible usage. These limitations of legally prescribed opioids appeared to be negatively impacting people’s quality of life.

#### Negative Impact of Opioids: The Risks and Harms of Opioid Misuse and Abuse

A second subtheme is the *risks and harms of opioid misuse and abuse*. While numerous participants explained the utility and importance of opioids, others expressed the danger of opioids. Participants reported that opioids have changed their loved one’s personalities, or they have experienced loss caused by opioids (i.e., death of a loved one, removal of parental rights, reduced functioning) due to someone misusing or abusing opioids:

*I have a cousin that was in a severe car accident because of opioids, and it destroyed her life... she has a TBI [Traumatic Brain Injury] and needs a walker to walk and get around now, and she thinks a lot differently now... I’ve had many friends that have died due to opioid use too...*

This participant explained the extreme risks of opioid misuse have had directly on their loved ones’ physical health and mortality. Other participants report how the *risks and harms of opioid misuse and abuse* indirectly affect them through crime. For instance, one participant explained:

*I don’t use opioids; no one in my family uses them either. But people in the city I live in do. It drives people to do crazy things. Someone tried to break into my car and apartment, and they were on opioids, at least that’s what the police told me. It was scary*.

This participant described how they believe that crime in their community was linked to opioid misuse according to their local law enforcement. There is a stigma that substance use, particularly opioid use, is associated with criminal/violent behavior. However, it is important to recognize that the substance use does not cause criminal/violent behavior; instead, such behavior is more appropriately attributed to the stigmatization and criminalization of substance use disorders as well as the lack of access to substance use interventions, which is particularly true in rural communities (31). This participant’s association between substance use and increased criminal/violent behavior perpetuates the stigma of persons with substance use disorders as “dangerous” and dehumanizes them and reduces the urgency for their need for intervention services. Finally, one participant, who claims to be actively using opioids, summarizes how the *risks and harms of opioid misuse and abuse* have negative impacts across multiple levels of society:

*Opioids have ruined my life. I was introduced to them by a third party to manipulate me [to] sell. My family is disappointed in me. I’m not a productive member of the community. Opioids have a very strong and negative impact on community*.

While this participant explained the negative impact, opioids have had on the community, they also underscored how using opioids negatively impacted their life, their family’s life, and strained their family relationships.

### Resiliency

The last overarching theme encapsulates many participants’ attempts to avoid opioids and/or recover from opioid addictions through their *resiliency*. Resilience can defined in many ways, though when expressed as a component of Community Resiliency Theory, Folke and colleagues^16^ state that *resilience* is a degree to which the system is capable of self-organization, and the degree to which the system can build capacity for learning and adaptation.^16^ For this study, individuals, communities and families that are *resilient* can learn and adapt to crisis in a way that allows them to utilize their strengths to maintain a minimum standard of life. Eighteen participants’ responses expressed this overarching theme.

#### Resiliency: Physically Moving

The first subtheme of resiliency was Physically Moving. Some participants expressed the need or desire to physically move out of communities impacted by opioids to avoid negative impacts of opioid misuse or abuse.

*Participant 1: I’ve been stolen from. Stole from money from me, personal items, important items. And it’s upset me, and I know they have a problem, so I’ve moved away*.

*Participant 2: My family is fairly responsible with regard to opioid use. I have a nephew who had a serious drug problem, but he has since moved out of the area and is doing well…*

Both of these participants provided stories about how choosing to leave communities affected by opioid misuse and abuse. While this option is not feasible for everyone, it appears to be a helpful option for these individuals.

#### Resiliency: Active Avoidance of Opioids

The second subtheme to resiliency was Active Avoidance. Another way some individuals reported resiliency is by actively avoiding opioids even when prescribed opioids because of witnessing the devastating impact of opioid misuse. For instance, one participant explains their reasoning behind their avoidance:

*I won’t take opioids even if prescribed. There have been two occasions after surgery that I took a pill or half of a pill...hydrocodone. I didn’t like the way it made me feel. I felt like I was walking sideways. I couldn’t safely walk my dog. I’m also too afraid of becoming addicted. The opioid drug abuse in [state] is the highest in the country… I don’t want to become a slave to a drug. I don’t want to be the person stealing to support a drug habit. So now, I won’t even fill a prescription for an opioid if the doctor writes one. I will tear up the prescription, or I will tell the doctor not to write it*.

This participant reported actively avoiding opioids to prevent addiction both because they do not like the way it makes them feel and because of the negative consequences they observed in their community.

#### Resiliency: Seeking Treatment

The fourth subtheme was seeking treatment. Many participants who became addicted to opioids in the past reported being resilient by attempting to stop their usage by entering treatment. For example, some participants expressed the following:

*Participant 3: I had problems in the past. But I overcame the addiction and moved on to a recovery program on my own. I am getting back on track one day at a time*.

*Participant 4: It ruined my life... it sent me into a drug-addicted spiral that was a 15-year blur... I lost my children and still feel like I can’t get on my feet... my family lost trust in me, and they want to do things with me... the loss of my children was a breaking point...to see them with family members breaks my heart... I have been clean for four months, and I’m trying to get back on my feet, but it’s so hard....*

*Participant 5: I was on IV drugs for ten years, current clean and sober four months. It affected my mother badly. It affected my and my child’s life negatively. IV drug use resulted in hepatitis C for me*.

All of these participants evidenced resiliency by trying to reduce or terminate their opioid misuse or abuse, despite the challenges of treatment and maintenance post-treatment. Several participants explained how their opioid usage negatively impacted them and their family members, and the impact on their family motivated them to enter treatment.

### Study 2: Telephone Interview

Also, using the content analysis, Study 2 identified similar overarching themes with the addition of a fourth theme (Systemic Restrictions and Frustrations). Despite these similarities in theme categories, the telephone interview provided more nuanced detail about the participants’ perspective. This is likely because it is often easier to talk then type responses to open-ended questions. These four themes from the telephone interviews were: (1) Benefits of Opioid Use, (2) Negative Impacts of Opioid Use, (3) Resiliency, and (4) Systemic Restrictions and Frustration. Like with the online interview, within each theme, we highlight several subthemes, how these themes spanned multiple levels of society (i.e., individual, family, community), and we noted similarities in the participant’s responses. It is important to note that, at times, responses fell into more than one category.

### Benefits of Opioid Use

Similar to Study 1, some participants expressed how opioids have been beneficial for them, despite the current opioid crisis occurring in their communities. Of the 26 participants that completed the telephone interview, 4 participants’ responses expressed this overarching theme. For instance, one participant explained:

*Well when we get pain medicine, it helps us improve our quality of life, and to live a little bit less pain-free from the pain we are experiencing, and we can be more productive and be able to do things that we wouldn’t be able to do…But in our community, I guess we’ re, they’re having this big opioid crisis or whatever so I guess it’s getting pretty bad or whatever*.

Similar to Study 1, this participant expressed the utility of opioids to perform daily functions and improve quality of life. Further, this participant (in addition to others) recognized the opioid crisis occurring in their area despite their responsible usage.

### Negative Impact of Opioid Usage

Also similar to Study 1, some participants described how opioids have negatively impacted them both indirectly and directly. Twenty-three participants’ responses fell into this theme. However, these participants’ concerns were all categorized into the subtheme, *The Risks, and Harms of Opioid Misuse and Abuse*.

#### Negative Impact of Opioid Usage: The Risks and Harms of Opioid Misuse and Abuse

Participants on the phone interview expressed frustration with how opioids misuse and abuse have had negative impacts. For example, participants explained:

*Participant 6: … It’s raised the crime level. Everything has to do with drugs. If you’ve got a robbery, it’s drugs. If you’ve got a murder, it’s drugs. Most of the traffic things, drugs. My niece’s boyfriend got hit by a car. They arrested him for drugs. I mean, it’s everything…*

*Participant 7: They won’t talk to me…None of my (inaudible) wants to talk to me. My kids, my family, nobody. Because of all them pain pills*.

*Participant 8: …I feel like there’s no economy, so the drug problem is very large. I’ve watched so many people my age die early. I’ve just watched so many become addicted. They have gotten on a lot harder drugs. It just makes me sick. To see what the things that drugs have done to [County name]. I think they’re just must be a better, another, way than drugging everybody. We’ve got to figure out other ways to cope with problems that we’ve got to resolve problems without having to take pills*.

These participants exemplify the perception of how opioid misuse and abuse has impacted communities at the three levels of society: individual, family, and community. Participant 6 described that opioid misuse is linked to increased crime in their area. Participant 7, who was actively using opioids, reported how their usage negatively impacted their close relationships, presumably with friends or family members. Participant 8 expressed their anger at the severe impact of opioid misuse and death in their community. Further, this participant alluded to the potential cyclical nature of opioid misuse, whereby opioid misuse is linked to community problems but also that community problems (e.g., poverty, unemployment) are linked to opioids misuse.

### Resiliency

Participants in the phone interview also stated ways they are resilient. Three of the 26 participants’ responses fell into this theme. However, most of these examples were under the subtheme’s active avoidance. For example, some participants expressed the following:

*Participant 9: …But a lot of people make a lot of excuses they think is a good excuse to take it. I believe a lot of people could take Tylenol or Motrin, something like that, stay off the real hard stuff, and real strong stuff. Maybe it would give them a better chance for a better life. Pain medication ain’t good for nobody. Not if they get on it bad enough*.

*Participant 10: I try to avoid them, even if I go to the doctor because I know I have an addictive personality, and that scares me, and I don’t want to go there…*

All of these participants evidenced resiliency by actively avoiding opioids. Some feared that they will fall victim to addiction, so they chose to not take any opioids, while some, like Participant 9, expressed frustration about why individuals choose to use opioids rather than managing their pain with other, nonpharmacologic, methods. This frustration demonstrated that while some people can actively avoid opioid misuse and addiction, there may be little understanding of how addiction occurs and is maintained within portions of these underserved communities.

### Systemic Changes and Frustrations

Unlike Study 1, there was a new theme identified in the phone interviews. The last overarching theme in Study 2 was how participants synthesize why the opioid epidemic has caused systemic restrictions in communities impacted by the opioid crisis. Five of the 26 participants’ responses expressed systemic changes and frustrations. This theme emphasizes how their lives have been negatively impacted by government or larger social systems in ways that are out of their control. For instance, some participants expressed the following:

#### Participant 1

*I really wish that health insurance was a lot easier to get, and it was a lot cheaper and easier to get prescriptions instead of how it is done now because of drug addicts who have ruined it. It used to be you could go to a doctor, and they could prescribe medicine or a clinic which could prescribe you pain medicine. Today they have special pain clinics, and you have to pay an exorbitant fee for pain medication, and then it lasts a month, and then you gotta go back again and pay and so it’s made things a lot harder*.

#### Participant 2

*They took me off of opioids, and the FDA blocked me from any prescription meds period. Cause of some desk jockey in Washington DC, I can’t even get aspirin if I go to the hospital. I can’t even get anything for pain because I’m blocked, and that’s because of the opioid crisis in the state …which is out of hand because of idiots who go to doctors and get prescriptions and sell them on the streets. So it’s affected me that way because I don’t get any pain medication, so I get to suffer 24/7…That’s what happens to people in pain when government decides you don’t get prescription medicine for your pain because other people abused it. That’s how I feel*.

Both of these participants describe how governmental and industrial systems, including clinics and insurance agencies, have changed as a result of the opioid epidemic. Some individuals that may have been using responsibly are becoming frustrated with these systemwide changes that they perceived have negatively impacted them.

## DISCUSSION SUPPLEMENT

Although there is a risk of opioid misuse, it is clear from participants’ perspective that prescribed use of opioids is critical to their own and the family members’ quality of life. Among our telephone sample, this frustration was made clearer with the additional theme of systemic changes and frustrations. This theme discussed frustrations with the barriers to accessing prescribed pain medication and how those barriers were caused by changes in the governmental or larger systemic levels. One possible solution to the frustrations around policy changes and perceived limitation to prescriptions is to enhance medical provider sensitivity and knowledge about patient pain and how prescribed pain medications improve the quality of many patients’ lives. More specifically, providers can enhance patient-centered decision-making by both educating patients and family members about treatment and risk behaviors and training providers to communicate more effectively about pain treatment expectations and alternative options to pain coping (e.g., increased exercise, mindfulness relaxation techniques).

The next theme we identified is the negative impact of opioid usage, namely, risks and harms associated with the misuse and abuse of opioids. Some of our participants talked about increased crime and a depressed economy, both perceived to be associated with opioid misuse. This sentiment has been reflected in the previous qualitative literature on the impact of opioids.^17^ However, our findings highlighted indirect harmful effects on families and family relationships. For example, one of our participants highlighted how losing contact and trust with their family was one of the more devastating aspects of their addiction. Challenges of reentering society have been highlighted in past literature, including entering the workforce and finding a new identity.^8^ However, what was less emphasized is the frequent loss of supportive social networks such as parental support and contact with children. Also, some participants in our study discussed the negative impact on children, such as living without a washer and dryer, a potential reference to child neglect. Neglectful family environments can have long-term mental and physical health outcomes.^8^,^18^,^19^ Across this theme, our participants perceive the negative impact of opioids across multiple levels of society. When developing and implementing intervention combating the far-reaching negative impacts of the opioid crisis, we must think systemically. We must target *individuals* who are in recovery, those seeking recovery or currently using, and those who have been indirectly exposed to the rippling effects of opioid addiction. We must target *families* to systemically heal ruptured relational bonds, prevent generational trauma caused by addiction, and improve social support for individuals in recovery or seeking treatment. We must target *communities* as a whole by building or rebuilding healthcare, economic, and safety net infrastructures so that communities can be stronger in the future to withstand the impact of a crisis like the opioid epidemic.

REFERENCES SUPPLEMENT1
ClickIABasdenJABohannonJMAndersonHTudiverF
Opioid prescribing in rural family practices: a qualitative study
Substance Use & Misuse
2018
53
4
533
40
2885764310.1080/10826084.2017.13426592
DesveauxLSaragosaMKithulegodaNIversN
Understanding the behavioural determinants of opioid prescribing among family physicians: a qualitative study
BMC family practice
2019
20
1
1
12
3107713710.1186/s12875-019-0947-2PMC65111633
RiesRKrupskiAWestII
Correlates of opioid use in adults with self-reported drug use recruited from public safety-net primary care clinics
Journal of addiction medicine
2015
9
5
417
2642836110.1097/ADM.0000000000000151PMC46064644
HurstakEEKushelMChangJ
The risks of opioid treatment: Perspectives of primary care practitioners and patients from safety-net clinics
Substance abuse
2017
38
2
213
21
2839475210.1080/08897077.2017.1296524PMC55685225
FadanelliMCloudDHIbragimovU
People, places, and stigma: a qualitative study exploring the overdose risk environment in rural Kentucky
International Journal of Drug Policy
2019
102588
3175360310.1016/j.drugpo.2019.11.001PMC72316296
AntoniouTAla-LeppilampiKShearerDParsonsJATadrousMGomesT
“Like being put on an ice floe and shoved away”: A qualitative study of the impacts of opioid-related policy changes on people who take opioids
International Journal of Drug Policy
2019
66
15
22
3068565010.1016/j.drugpo.2019.01.0157
AllenSTGriebSMO’RourkeA
Understanding the public health consequences of suspending a rural syringe services program: a qualitative study of the experiences of people who inject drugs
Harm reduction journal
2019
16
1
1
10
3110933910.1186/s12954-019-0305-7PMC65282868
BehringerB
Listening to Voices in Appalachia: Gathering Wisdom from the Field about Substance Abuse Recovery Ecosystems
Journal of Appalachian Health
2020
2
3
10.13023/jah.0203.11PMC9138758357702129
MathisSMHagemeierNFosterKNBakerKPackRP
“It’s Took Over This Region”: Patient Perspectives of Prescription Drug Abuse in Appalachia
Substance Use & Misuse
2020
55
1
37
47
3152617710.1080/10826084.2019.1654514PMC691798110
DriscollMAKnobfMTHigginsDMHeapyALeeAHaskellS
Patient experiences navigating chronic pain management in an integrated health care system: A qualitative investigation of women and men
Pain Medicine
2018
19
suppl_1
S19
S29
3020300910.1093/pm/pny13911
WiederRI
Evaluating what makes a US community urban, suburban or rural In Decoded
Pew Research Center
2019
12
LincolnYSGubaEG
Naturalistic inquiry
Newberry Park CA
Sage
1985
13
CreswellJW
Educational research: Planning, conducting, and evaluating quantitative
Prentice Hall
Upper Saddle River, NJ
2002
14
CreswellJWPothCN
Qualitative inquiry and research design: Choosing among five approaches
Sage publications
2016
15
CorbinJStraussA
Basics of qualitative research: Techniques and procedures for developing grounded theory
Sage publications
2014
16
FolkeCCarpenterSElmqvistTGundersonLHollingCSWalkerB
Resilience and sustainable development: building adaptive capacity in a world of transformations
AMBIO: A journal of the human environment
2002
31
5
437
40
10.1579/0044-7447-31.5.4371237405317
ThomasNvan de VenKMulrooneyKJ
The impact of rurality on opioid-related harms: A systematic review of qualitative research
International Journal of Drug Policy
2019
102607
3186478710.1016/j.drugpo.2019.11.01518
NormanREByambaaMDeRButchartAScottJVosT
The long-term health consequences of child physical abuse, emotional abuse, and neglect: a systematic review and meta-analysis
PLoS Med
2012
9
11
e1001349
2320938510.1371/journal.pmed.1001349PMC350796219
BrownDSFangXFlorenceCS
Medical costs attributable to child maltreatment: A systematic review of short-and long-term effects
American journal of preventive medicine
2011
41
6
627
35
2209924110.1016/j.amepre.2011.08.013

## Figures and Tables

**Table 1:** Categories Formed from Content Analyses

	Benefits of Opioid Usage	Negative Impacts of Opioid Usage	Resiliency/ Systemic Frustrations
Self	Benefits of opioid usage (e.g., improves quality of life, able to participate in life)	The limitation of access to opioids The risks and harms of opioid misuse and abuse	Physically moving Active avoidance of opioids Seeking treatment
Family/Social	Benefits of opioid usage (e.g., Improves family members quality of life)	The risks and harms of opioid misuse and abuse (e.g., lost contact with family/friends)	Physically moving
Community		The risks and harms of opioid misuse and abuse (e.g., crime rates increase)	Systemic changes and frustrations
